# Low-dose mistletoe lectin-I reduces melanoma growth and spread in a scid mouse xenograft model

**DOI:** 10.1038/sj.bjc.6604106

**Published:** 2007-11-20

**Authors:** A Thies, P Dautel, A Meyer, U Pfüller, U Schumacher

**Affiliations:** 1 Zentrum für Experimentelle Medizin, Institut für Anatomie II: Experimentelle Morphologie, Universitätsklinikum Hamburg Eppendorf, Martinistrasse 52, D-20246 Hamburg, Germany; 2 Institut für Phytochemie, Fakultät für Biowissenschaften, Private Universität Witten Herdecke gGmbH, Stockumer Strasse 10, 58448 Witten, Germany

**Keywords:** malignant melanoma, MV3, mistletoe lectin, metastasis, dendritic cells

## Abstract

This study investigates the effects of mistletoe lectin-I (ML-I) on melanoma growth and spread *in vivo*. The human melanoma cell line MV3 was xenografted into severe combined immunodeficient mice and vehicle solution or purified ML-I was administered at 30, 150 and 500 ng per kg body weight (20 mice per group) daily. After 19 days, mice were killed, primary tumours (PTs) and lungs were dissected out, and tumour weights, number of lung metastases (LMs), number of tumour-infiltrating dendritic cells (DCs), and apoptosis rates in the melanoma cells and in the DCs were assessed. A 35% reduction of PT weight (*P*=0.03) and a 55% decrease in number of LMs (*P*=0.016) were evident for low-dose ML-I (30 ng kg^−1^) treatment but not for higher doses. Mistletoe lectin-I increased apoptosis rates in the melanoma cells of PTs at all doses, while no induction of apoptosis was noted in the LMs. Low-dose ML-I significantly increased the number of DCs infiltrating the PTs (*P*<0.0001) and protected DCs against apoptosis, while higher doses induced apoptosis in the DCs (*P*<0.01). Our results demonstrate that low-dose ML-I reduced melanoma growth and number of metastases *in vivo*, primarily due to immunomodulatory effects.

Despite intensive research, no curative treatment exists for malignant melanoma once it has spread to distant sites (Eigentler *et al*, 2003). Conventional chemotherapy or combination of radio- and chemotherapy have been disappointingly ineffective and have not led to any considerable prolongation of survival. The hope to improve survival expectations for melanoma patients thanks to immunotherapies has either not yet been fulfilled (Edler, 2004). Thus, it is not astonishing that about 40% of tumour patients turn to alternative treatment options such as aqueous mistletoe extracts. In Germany, more than 30 million Euro are spent yearly with increasing tendency (Steuer-Vogt *et al*, 2001). However, there are considerable discrepancies between this widespread usage and the few numbers of clinically controlled trials on the effect of mistletoe extracts in cancer therapy, which, moreover, report controversial results (Kienle *et al*, 2003; Edler, 2004). One obvious explanation is that the precise mode of action of aqueous mistletoe extracts is still unclear. The main therapeutic components of mistletoe extracts are the three mistletoe lectins (MLs) ML-I, -II, -III (Büssing *et al*, 1996). However, mistletoe extracts contain further numerous low molecular weight compounds such as viscotoxins, polysaccharides, amino acids and phenols, which might show additional or even reverse biological effects on tumour cells (Beuth, 1997). Hence, the effect of purified MLs on melanoma cells in general and on disseminated ones in particular is of considerable clinical interest.

Of the three MLs, ML-I is the best investigated and consists of an A-chain having a strong cytotoxic effect due to its ribosome-inactivating properties (Rip type II) and a B-chain carrying the carbohydrate-binding site (Barbieri *et al*, 1993; Langer *et al*, 1996). A prerequisite for the internalisation and the cytotoxic activity of ML-I is its binding to the target cell surface mediated through the lectin binding domain of the B-chain. As both metastatic primary malignant melanomas and their metastases express particularly high numbers of ML-I binding sites, malignant melanoma cells represent an ideal target for ML-I cytotoxic therapy (Thies *et al*, 2001, 2007a). Previous *in vitro* experiments have already demonstrated a highly significant antiproliferative effect of ML-I on malignant melanoma cells, which is due to the induction of apoptosis (Thies *et al*, 2005). In addition to its cytotoxic effect, ML-I might have further impact on tumour growth and metastases through its stimulation of the immune system, raising the number and the activity of NK cells, dendritic cells (DCs) and granulocytes (Hajto *et al*, 1997; Pryme *et al*, 2006). Furthermore, antiangiogenic effects have been described for the Korean ML-I (Park *et al*, 2001).

However, before purified ML-I can be applied in humans, extensive preclinical analyses have to be performed. We have already established a clinically relevant human melanoma xenograft scid mouse model, in which the effects of drugs on melanoma growth and spread can be analysed (Thies *et al*, 2007b).

The first aim of this study is to analyse the potential therapeutic effects of purified ML-I on melanoma cell growth and spread in our established human melanoma xenograft scid mouse model. The second aim is to analyse the mechanisms responsible for ML-I effects on melanoma *in viv*o by assessing apoptosis rates, the number of infiltrating DCs, and vascular counts in primary melanomas and their spontaneous lung metastases (LMs).

## MATERIALS AND METHODS

### Cell culture

The human melanoma cell line MV3 was established from a metastatic melanoma lymph node (Edward, 2001). This cell line has shown both a 100% subcutaneous tumour engraftment and a 100% spontaneous LM rate within 20 days, when engrafted into scid mice (Thies *et al*, 2007b).

MV3 cells were cultured under standard cell culture conditions (37°C, 100% relative humidity, 5% CO_2_) in RPMI medium (Gibco, Paisley, Scotland), supplemented with 10% heat-inactivated fetal bovine serum (Gibco), 2 mM L-glutamine (Gibco), 100 U ml^−1^ penicillin and 100 *μ*l ml^−1^ streptomycin (Gibco). The cells were tested for the presence of mycoplasma using the PCR-based VenorGeM Mycoplasma Detection Kit (Minerva Biolabs GmbH, Berlin, Germany). For injection, mycoplasma-free melanoma cells were harvested by trypsinisation, tested for viability (viability>95%) and were adjusted to a concentration of 5 × 10^6^ viable cells per 1 ml medium.

### ML-I

Mistletoe lectin-I was isolated from leaves of the European mistletoe (*Viscum album*) harvested from poplar (40%), apple tree (30%) and red oak (30%) between November and February. Purification was performed using affinity chromatography according to Eifler *et al* (1994). The ML-I stock solution contained 963 *μ*g ML-I per ml of solution, and about 1% ML-III and less than 0.5% ML-II.

To avoid erroneous dosage of ML-I through surface adsorption effects (Cleland *et al*, 1993), polysterol tubes (Greiner, Frickenhausen, Germany) were used for the preparation of the different ML-I dilution series and glass pistons were used for injection.

### Animals

The methodology for carrying out the experiment was consistent with the UKCCCR guidelines for the welfare of animals in experimental neoplasia (Workman *et al*, 1997). The experiment was supervised by the institutional animal welfare officer and approved by the local licensing authority (Behörde für Soziales, Gesundheit, Familie, Verbraucherschutz; Amt für Gesundheit und Verbraucherschutz, Hamburg, Germany, project no. F1 8/01).

Pathogen-free male balb/c severe combined immunodeficient scid/scid mice aged 9–14 weeks were housed in filter top cages and were provided with sterile water and food *ad libitum*. All manipulations were carried out aseptically inside a laminar flow hood. Before injection of the melanoma cells, mice were weighted and grouped (adjusted to a mean body weight of 20 g (range: 18.5–21.0 g) into three treatment groups and one control group (20 mice per group). One million MV3 cells (in 200 *μ*l medium) were injected subcutaneously between the scapulae of each scid mouse. Mice received intraperitoneal injections of 200 *μ*l PBS containing 30, 150 or 500 ng ML-I per kg of body weight once daily for next 19 days. Control mice were treated the same way, receiving vehicle solution only.

### Vitality score

All mice were inspected daily and the overall clinical condition, including appearance, posture, behaviour and physiological responses, as well as food and water intake, was assessed. Each position was rated from 1 to 3 points, resulting in a maximum of 12 points for animals with no vital detraction.

### Histology

On day 20, the mice were killed by cervical dislocation. The tumours were excised within their capsule, weighed and immediately fixed in 4% neutrally buffered formalin. The lungs of all animals were dissected out *en bloc*, and fixed in 4% neutrally buffered formalin for 48 h. Thereafter, the lungs were cut under a dissecting microscope into 1 mm-thick slices, which were spread randomly over a glass slide and then embedded in 4% warm liquefied agar. Slices were pressed down gently with a glass piston to avoid floating of the slices within the agar during cooling. The solidified agar blocks were then routinely processed for wax histology and were serially sectioned. The number of LMs was analysed using the simplified quantitative method standardised in our laboratory as described by Jojovic and Schumacher (2000). Briefly, every tenth section of each lung was retained (the total number of sections per lung was evaluated), and 10 sections from the middle of the block were stained with H&E, and the number of LMs was counted under 100 magnification field. The mean numbers of LMs in the 10 sections for each lung (mean value_10_) were calculated. This mean value_10_ minus 20% was multiplied by the total number of serial sections of the respective lung in order to estimate the total number of LMs. Furthermore, the size (tumour cells per metastasis) and the anatomical site of LMs (intravasal *vs* extravasal; pulmonary artery, bronchial vessels, intraseptal tissue) were recorded.

### BSA-I histochemistry

For evaluation of the number of tumour-infiltrating DCs, paraffin sections (5 *μ*m) were processed for BSA-I histochemistry (Honda *et al*, 1989; Thiele *et al*, 2000) using an avidin–biotin alkaline phosphatase staining technique as has been reported previously (Thies *et al*, 2001). Briefly, trypsinised tissue sections were incubated with 10 *μ*g ml^−1^ biotinylated BSA-I (Sigma, Steinheim, Germany) followed by an incubation with an avidin–alkaline phosphatase complex (Vectastain, Vector, ABC kit, Burlingame, CA, USA). Enzyme reactivity of the complex was visualised using naphthol-AS-bisphosphate as a substrate, and hexatozised New Fuchsin was used for simultaneous coupling. Negative controls were treated the same way preincubating BSA-I with its nominal sugar *α*-D-galactose. The number of DCs was taken as the mean of the DC count in five high-power fields (magnification × 400) in slides of PTs and LMs of each mouse.

### Apoptotic rates and dimension of ulceration

Apoptotic rates were determined on H&E-stained sections of primary tumours (PTs) and LMs of each animal, as described by Kerr *et al* (1972), who have set the most secure and exact methods, when diverse methodological approaches to analyse apoptotic rates are compared (Hall, 1999). Apoptotic rate was taken as the mean of the apoptotic count in four high-power fields (magnification × 400) in the vital tumour area of each PT and of each LM.

Apoptotic rates within the tumour-infiltrating DCs were determined in BSA-I-stained slides, counterstained with H&E, according to the same criteria described above.

The dimension of ulcerations in the PTs was evaluated microscopically according to the morphologic criteria described by Kerr *et al* (1972) in H&E-stained sections of PTs and measured digitally using the program AxioVision (Zeiss, Oberkochen, Germany).

### Collagen type IV immunohistochemistry

For antigen retrieval, slides were treated with 0.04% protease XXIV (Sigma). Nonspecific binding was blocked by 10% normal rabbit serum. This was followed by an overnight incubation with the 1 : 200 diluted goat anti-collagen type IV antibody. Then, sections were incubated with biotinylated rabbit anti-goat antibody for 40 min, followed by an incubation with an avidin–alkaline phosphatase complex (Vectastain, Vector). Enzyme reactivity of the alkaline phosphatase was visualised as described above. Negative controls were treated the same way except replacing the primary antibody by the isotype-matched IgG. The number of tumour-infiltrating vessels per 0.5 mm^2^ was assessed as mean of vascular counts in five fields of view adjusted to 0.5 mm^2^ in vital tumour tissue.

### Statistical analyses

For all variables, one-way ANOVA tests followed by a Tukey test were performed to ascertain statistical differences between the different treatment groups, using Graph Pad Prism Version 4 (Intuitive Software for Science, San Diego, CA, USA). *P*<0.05 was considered statistically significant. Graphs show mean and SEM. Furthermore, correlation analyses between tumour weights, number of LMs, dimension of necrotic tumour areas and vascular counts in the different treatment groups were performed using the Spearman rank correlation. Again, *P*<0.05 was considered statistically significant.

## RESULTS

### Tolerability of ML-I treatment

Tumour growth and ML-I treatment at all three doses had no negative effect on vitality, behaviour and physiological responses, appearance or food and water habits of any of the animals. All animals reached vitality scores of 11–12 out of 12 during and at the end of the experiment. At necropsy, no treatment-related lesions could be observed.

### Primary tumours

**Figure 1:**
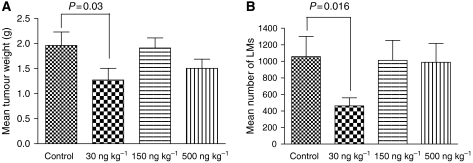
(**A**) Mean tumour weights. Low-dose ML-I (30 ng per kg body weight) daily for 19 days significantly reduced the weight of tumour nodules grown from subcutaneously injected MV3 human melanoma cells, while higher doses had no significant effect. (**B**) Mean number of lung metastases. Mice treated with low-dose ML-I (30 ng per kg body weight) showed significantly less spontaneous LMs from subcutaneously injected human melanoma cells than the untreated control mice. The mean number of LMs in the 30 ng kg^−1^ treatment group was reduced by more than 50%.

The mean tumour weight in the control group (PBS-only) was 1.96±0.23 g, compared to 1.27±0.23, 1.9±0.2 and 1.5±0.18 g in the 30, 150 and 500 ng kg^−1^ group, respectively. The statistical analyses showed a significant reduction of the mean tumour weight in the group treated with 30 ng kg^−1^ compared to the control group (*P*=0.03), while mean tumour weights of the groups treated with 150 or 500 ng kg^−1^ did not significantly differ from that of the control group (*P*>0.05; Figure 1A). Mean tumour weights of mice treated with 150 ng ML-I per kg body weight were significantly higher than those of the mice treated with low-dose ML-I (30 ng kg^−1^; *P*=0.04).

### Number of lung metastases

All except two mice showed LMs. The two mice without LMs had a small PT (<0.5 g) and belonged to the 150 ng kg^−1^ group and control group. These mice were excluded from the analyses.

The mean number of LMs in the control group was 1057±244, compared to 461±99, 1012±241 and 989±228 in the 30, 150 and 500 ng kg^−1^ group, respectively.

A significant reduction of LMs was detected in the 30 ng kg^−1^ group compared to the control group (*P*=0.016), while in the two groups treated with higher ML-I doses (150 and 500 ng kg^−1^), no significant reduction of the number of LMs was evident (Figure 1B). The mean number of LMs of mice treated with 150 and 500 ng ML-I per kg body weight was significantly higher than that of the mice treated with low-dose ML-I (30 ng kg^−1^; *P*=0.02).

### Size of lung metastases

Lung metastases of the control group had a mean size of 36 cells (±6.4). Mistletoe lectin-I treatment at 30, 150 and 500 ng kg^−1^ did not significantly alter the mean size of the LMs, which was 36±7.9, 37±3.5 and 35.2±6.1 cells per metastasis, respectively (*P*>0.05, Supplementary Information 1).

### Induction of apoptosis in the melanoma cells

**Figure 2:**
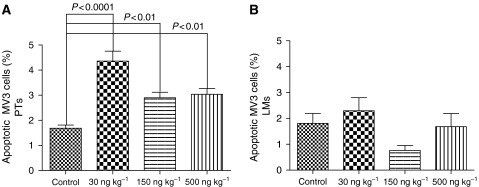
(**A**) Percentage of apoptotic melanoma cells in the PTs. Mistletoe lectin-I treatment at any concentration induced apoptosis in the melanoma cells of the PTs. (**B**) Percentage of apoptotic melanoma cells in the LMs. Mistletoe lectin-I treatment had no significant effect on the apoptotic rate in the lung metastases.

Apoptotic rates in the PTs were significantly increased in all three treatment groups compared to the control group, which showed an apoptotic rate of 1.68%. Low-dose ML-I treatment (30 ng ML-I per kg body weight) increased apoptosis rates by a factor of 2.6 (4.36% apoptotic melanoma cells; *P*<0.0001), while 150 and 500 ng kg^−1^ increased the number of apoptotic tumour cells by a factor of 1.7 and 1.8, respectively; *P*<0.01 (Figure 2A). Mistletoe lectin-I concentrations of 150 and 500 ng kg^−1^ treatment resulted in significantly lower apoptosis rates compared to 30 ng kg^−1^ (*P*<0.001 and *P*<0.01, respectively). Apoptosis rates in between 150 and 500 ng ML-I per kg did not differ significantly (*P*>0.05).

In the LMs, no significant changes of apoptotic rates through ML-I treatment were noted (*P*=0.09; Figure 2B). Lung metastases of the control group showed apoptotic rates of 1.8%. Apoptotic rates in the treated groups ranged from 0.8% (150 ng kg^−1^ group) to 2.3% (30 ng kg^−1^ group).

### Number of infiltrating DCs

**Figure 3:**
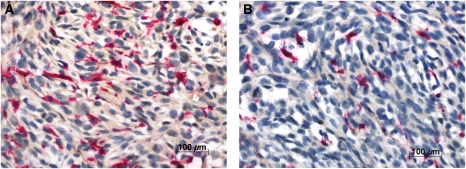
BSA-I lectin histochemistry. Tumour-infiltrating DCs were characterised thanks to their high BSA-I binding capacity; (**A**) PTs of mice treated with 30 ng ML-I per kg body weight showed high numbers of DCs, while PTs of untreated control mice (**B**) were less infiltrated by DCs.

**Figure 4:**
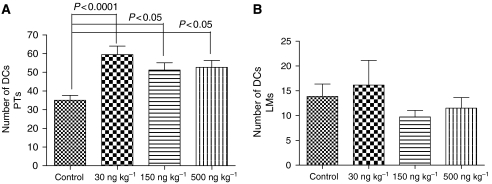
Mean number of infiltrating DCs in primary melanomas. Mistletoe lectin-I treatment significantly enhanced the number of DCs infiltrating the primary melanomas (**A**), but had no influence on DC number infiltrating the LMs (**B**).

Dendritic cells showed homogenous intensive cytoplasmic as well as membranous binding of the lectin BSA-I (Figure 3) and were easy to detect in the PTs and LMs, as the melanoma cells did not bind this lectin. The DC count in the control group was 35 (±2.6). Mistletoe lectin-I treatment at doses of 30, 150 and 500 ng ML-I per kg body weight significantly raised the number of infiltrating DCs (59.5±4.5, *P*<0.0001; 51. 2±3.9, *P*<0.05; and 52.7±3.7, *P*<0.05, respectively; Figure 4A).

The DC count in the LMs of the control group was 13.9 (±2.5). In the LMs, the number of infiltrating DCs was not significantly influenced by ML-I treatment (*P*>0.05, for all ML-I doses; Figure 4B).

### Induction of apoptosis in tumour-infiltrating DCs

**Figure 5:**
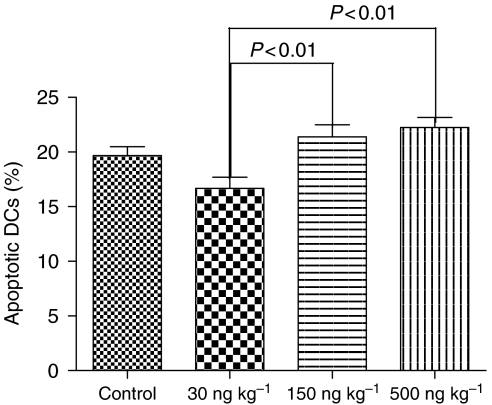
Percentage of apoptotic DCs in primary melanomas. Low-dose ML-I (30 ng kg^−1^) protected DCs against apoptosis, while doses higher than 30 ng per kg body weight significantly induced apoptosis in the DCs infiltrating the primary melanomas.

The ANOVA analysis of the percentage of apoptotic DCs in the total number of DCs infiltrating the primary melanomas (Figure 5) revealed significant differences in the apoptotic rates in between the different treatment groups (*P*=0.001). Low-dose ML-I treatment (30 ng per kg body weight) reduced the apoptotic rate compared to the control group (15.74% apoptotic DCs *vs* 18.59%), which, however, did not reach statistical significance (*P*>0.05). When the apoptosis rates in between the three ML-I treatment groups were calculated with the Tukey post-ANOVA test, a significant increase in apoptotic rates within the DCs in the PTs of the 150 and 500 ng kg^−1^ groups (21.39 and 22.23% apoptotic DCs) compared to the 30 ng kg^−1^ group (15.74% apoptotic DCs) became evident (*P*<0.01; Figure 5).

### Viable DCs

**Figure 6:**
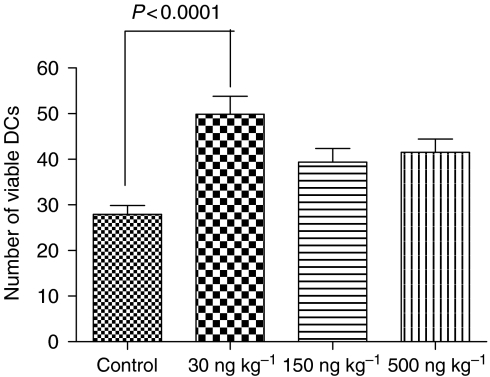
Number of viable DCs. Low-dose ML-I significantly enhanced the number of viable DCs infiltrating the PTs, whereas higher ML-I abrogated this effect.

When the number of viable DCs was calculated with the Tukey post-ANOVA test, significantly increased number of PT-infiltrating viable DCs was found in the 30 ng kg^−1^ group compared to the control (*P*<0.0001) but not any other groups (Figure 6).

### Tumour vascularisation

Tumour vessels were highlighted using collagen type IV immunohistochemistry (Supplementary Information 2a: PT of a mouse treated with 500 ng ML-I per kg; Supplementary Information 2b: PT of the control group). The vascular count (mean number of vessels per 0.5 mm^2^) in vital tumour mass of melanomas treated with ML-I at a concentration of 500 ng kg^−1^ was significantly increased compared to the control group (42.0±1.7 and 32.4±1.6, respectively; *P*=0.001) and to the 30 and 150 ng kg^−1^ group (34.74±1.3 and 35.1±1.6, respectively, *P*<0.05; Supplementary Information 2c).

### Necrotic tumour area

Primary tumours of all groups showed large necrotic areas (Supplementary Information 3) of about 35% of the overall tumour mass. Statistical analyses revealed no significant differences in the dimension of necrotic tumour area between the different groups (*P*>0.05; Supplementary Information 4).

### Correlation analyses

The results of the correlation analyses are summarised in Table 1. The rank correlation (Spearman) showed a significant positive correlation between the weight of the PT and the number of LMs in the control group and in all three treatment groups. The number of tumour vessels was neither correlated with the dimension of necrosis within the PTs nor with the number of LMs (*P*>0.05). The number of tumour vessels was positively correlated with the weight of the PT in the control group but not in the treatment groups.

## DISCUSSION

The present study was designed to investigate the potential antiproliferative and antimetastatic effects of purified ML-I in a human melanoma scid mouse xenograft model.

Purified ML-I was used, as a strong antiproliferative effect on a number of human melanoma cell lines, due to the induction of apoptosis, has already been demonstrated *in vitro* (Thies *et al*, 2005). The human melanoma cell line MV3 was used to model a targeted therapy *in vivo*, since MV3 cells expressed high numbers of ML-I-binding sites and proved to be ultrasensitive to ML-I cytotoxicity *in vitro* (Thies *et al*, 2005).

Our previously obtained *in vitro* results can now be extended to *in vivo*, as our results demonstrate a significant antitumorigenic and an antimetastatic effect of purified ML-I on malignant melanoma. Purified ML-I administered at 30 ng per kg body weight daily for 19 days reduced the mean tumour weight by 35% (*P*=0.031). The number of LMs was reduced by even 56% (*P*=0.015) compared to the control mice. Both beneficial effects, antiproliferative and antimetastatic, however, were only achieved at low-dose ML-I (30 ng per kg body weight), but not at higher doses (150 and 500 ng kg^−1^). Therefore, counterproductive mechanisms of ML-I effects at higher doses (150 and 500 ng ML-I per kg) abrogate the significant beneficial ML-I effects exerted at low-dose treatment (30 ng kg^−1^).

This significant stronger effect of low-dose ML-I on tumour growth and spread and reduction or even abrogation of its beneficial effects at higher doses has already been described in different tumour entities. Schumacher *et al* (2000) have shown similar effects of recombinant ML-I (rML-I) in a human ovarial carcinoma xenograft scid mouse model. Alike our results, only low-dose rML-I (30 ng kg^−1^) significantly reduced tumour growth, while higher doses (150, 500 ng kg^−1^) lost this effect. Weber *et al* (1998) showed that Lektinol®, a towards ML-I content standardised mistletoe extract, inhibited lung colonisation of i.v. injected melanoma cells at a 30 ng ML-I per kg body weight equivalent dosage, while higher doses were less effective. Hence, these results emphasise the need to answer the question for the mechanisms underlying dose-dependent differential ML-I effects.

One such mechanism might be found in the immunomodulatory effect of ML-I. In addition to its cytotoxic effect, ML-I is known to have a broad influence on the immune system, as increased levels of interleukins, TNF-*α*, interferon-*γ* and granulocyte–monocyte colony-stimulating factor, as well as an increased activity of macrophages and NK cells have been shown *in vitro* and *in vivo* (Hajto *et al*, 1989, 1997). These immunostimulatory effects are exerted at low-dose ML-I (1–10 ng ml^−1^), whereas higher doses (above 100 ng ml^−1^) abrogate these effect by inducing apoptosis in the leucocytes (Büssing *et al*, 1997). Hajto *et al* (2005) reviewed the effects of ML-I *in vitro* and *in vivo*, demonstrating differential susceptibility of different cell types and even of leucocyte subpopulations towards ML-I cytotoxicity.

To evaluate the cytotoxic effect of ML-I on malignant melanoma *in vivo*, we assessed apoptotic rates of the malignant melanoma cells, demonstrating a significant direct cytotoxic effect of ML-I on malignant melanoma cells in the PTs. The strongest cytotoxic effect was noted at low-dose ML-I (30 ng kg^−1^), where apoptosis rates were increase by a factor of 2.6 over the vehicle control (*P*<0.0001). Primary tumours in the groups with higher doses also showed increased apoptosis rates (factor 1.7 (150 ng kg^−1^) and 1.8 (500 ng kg^−1^); *P*<0.01); however, apoptosis rates did not increase in parallel to increased ML-I concentrations as one would have expected from our *in vitro* data (Thies *et al*, 2005). As no significant reduction in tumour weight was noted in the two groups with higher ML-I doses, the direct cytotoxic effect of ML-I seems to play only a minor role in its antitumorigenic effect *in vivo.* Thus, these results demonstrate the importance of *in vivo* models.

To elucidate the significance of immunomodulatory effects of ML-I on melanoma growth and spread, the number of tumour-infiltrating DCs was analysed. Mistletoe lectin-I treatment significantly increased the total number of DCs in the PTs of all treatment groups. However, DCs express high numbers of ML-I-binding sites (Thies *et al*, 2007b), which is not only the prerequisite for activation by ML-I but also for internalisation of the lectin followed by apoptosis induction in the DCs (see above). We therefore analysed apoptosis rates within the DCs in the different treatment groups, demonstrating that low-dose ML-I (30 ng kg^−1^) protected DCs against apoptosis (apoptosis rate reduced by about 16% compared to control), while higher ML-I doses (150 and 500 ng kg^−1^) significantly increased apoptosis rates in the DCs (*P*<0.01). Analysing the number of viable DCs, we could demonstrate that viable DCs were increased in the 30 ng ML-I per kg group but not in the two groups receiving higher ML-I doses. These results would attribute the antimelanoma effects of ML-I at low dose (30 ng kg^−1^) to the number of infiltrating DCs, and would suggest that the stimulation of the immune system plays the prominent role in its antimelanoma effect. Thus, low-dose ML-I both raised the number of DCs and simultaneously protected them against apoptosis.

In contrast to the above discussed effects of ML-I on PTs, in the LMs, neither a significant induction of apoptosis in the melanoma cells nor an increased number of infiltrating DCs at any ML-I concentration was evident. Hence, the prominent reduction of the number of LMs in the group treated with low-dose ML-I (30 ng kg^−1^) can neither be attributed to a direct cytotoxic effect on the tumour cells in the LMs nor to enhanced DC infiltration. As correlation analyses revealed a highly significant positive correlation between the weight of the PTs and the number of corresponding LMs in all groups, the significant reduction of LMs at low-dose ML-I (30 ng ML-I per kg) seems to be the consequence of successful reduction of the PT mass due to ML-I treatment. Additionally, a cytotoxic effect on circulating melanoma cells in the bloodstream, which are more prone to apoptosis induction than cells within the PT (Weber *et al*, 1998), could have attributed to the prominent reduction of the number of LMs. Especially, the reduction of the number of LMs but not of their size underlines the considerably stronger cytotoxic effect on circulating tumour cells, while in established metastases cytotoxic effects of ML-I seem to play a minor role.

As ML-I both induces apoptosis in tumour cells and simultaneously enhances the number of PT-infiltrating DCs, adjuvant ML-I treatment could have important impact in future melanoma therapy. Upon phagocytosis of apoptotic melanoma cells, DCs induce a strengthened specific immune response (Shaif-Muthana *et al*, 2000). This effect is already used for tumour vaccination (Ehlken *et al*, 2004), and melanoma patients vaccinated with mature DCs showed significant tumour regression (Hersey *et al*, 2004; Rescigno *et al*, 2004). Furthermore, the tumour-induced reduction of DC number and activity in the sentinel lymph node, which increases the risk for metastasis (Vuylsteke *et al*, 2004), was diminished by preoperative local injection of granulocyte–macrophage colony-stimulating factor, significantly improving the patient's prognosis (Vuylsteke *et al*, 2004). As scid mice lack T- and B lymphocytes, specific antimelanoma immune response upon ML-I treatment could not be analysed in our model system. However, local injection of low-dose ML-I might have similar beneficial effects as local injection of granulocyte–macrophage colony-stimulating factor and, furthermore, it would simultaneously induce apoptosis in the melanoma cells.

To analyse whether the European ML-I acts as an antiangiogenic agent, as has been discussed for Korean ML-I (Yoon *et al*, 1995; Park *et al*, 2001), we quantified the number of tumour-infiltrating vessels. However, no antiangiogenic effect of the European ML-I could be shown in our model. High-dose ML-I (500 ng kg^−1^) even increased the vessel density in the PTs compared to the controls (*P*>0.001). To elucidate this discrepancy, we analysed whether hypoxia in the necrotic and subnecrotic tumour area, being a strong proangiogenic trigger (Moeller *et al*, 2004), might have influenced our vascular count data. However, all groups showed comparable dimension of necrosis within the PTs and no correlation between diameter of necrosis and tumour vessels was evident. Thus, in our model no antiangiogenic effect of European ML-I was evidenced; however, differences in the effects on various tumour cells between Korean and European ML-I have already been shown (Yoon *et al*, 1999). Further correlation analyses revealed no correlation between vascular density and the number of LMs, or the weight of the PT, which shows that the period of 20 days of tumour growth and ML-I treatment might be too short to sufficiently model the complex mechanisms of the effects on vascularisation. However, the size of the PTs in the control group limited the duration of the experiment.

ML-I treatment had no negative influence on the vitality, behaviour and physiological responses, appearance, or food and water habits of the animals at any dosage, underlining the excellent tolerance of ML-I treatment. Studies on the quality of life of tumour patients are in accordance with our findings. Breast cancer patients treated with ML-I showed elevated *β*-endorphin levels (Heiny and Beuth, 1994), which would also be beneficial for melanoma patients. Furthermore, adjuvant ML-I therapy improved the tolerance of radio- and chemotherapy for different tumour entities (Kienle *et al*, 2003; Bock *et al*, 2004).

In conclusion, low-dose purified ML-I reduced melanoma growth and number of metastases in a xenograft model. The enhancement of DC infiltration and apoptosis induction in the melanoma cells seem to play the key role for these observed effects.

## Figures and Tables

**Table 1:** Spearman rank correlation analyses

**Variables**	**Treatment group**	***r***	***P***
TW, LMs	Control	0.6	0.005^*^
	30 ng/kg	0.8	<0.0001^*^
	150 ng/kg	0.7	0.0005^*^
	500 ng/kg	0.7	0.002^*^
VC, TW	Control	0.4	0.04^*^
	30 ng/kg	0.2	0.3
	150 ng/kg	−0.06	0.4
	500 ng/kg	−0.2	0.2
VC, LMs	Control	0.2	0.18
	30 ng/kg	0.1	0.30
	150 ng/kg	0.1	0.42
	500 ng/kg	−0.1	0.33
VC, Nec	Control	−0.1	0.29
	30 ng/kg	0.3	0.10
	150 ng/kg	−0.02	0.47
	500 ng/kg	0.1	0.39

LMs=number of lung metastases; Nec=dimension of necrosis; TW=tumour weight; VC=vascular count. ^*^Significant correlation.
