# Supplementary material for: Low-dose mistletoe lectin-I reduces melanoma growth and spread in a scid mouse xenograft model
Source: Br J Cancer. 2007 Nov 20;98(1):106–12. doi: 10.1038/sj.bjc.6604106 (PMC2359693; doi:10.1038/sj.bjc.6604106)
Supplement: Supplementary Figures [file 6604106x1.doc]

**Supplementary Figure 1**

**Supplementary Figure 2**

**a**

**b**

**Supplementary Figure 2c**

# Supplementary Figure 3

**Supplementary Figure 4**
