# Supplementary material for: Low-dose mistletoe lectin-I reduces melanoma growth and spread in a scid mouse xenograft model
Source: Br J Cancer. 2007 Nov 20;98(1):106–12. doi: 10.1038/sj.bjc.6604106 (PMC2359693; doi:10.1038/sj.bjc.6604106)
Supplement: Supplementary Figures Legend [file 6604106x2.doc]

**Supplementary Figure 1:** Mean size of metastases. ML-I treatment had no influence on the mean size of lung metastases.

**Supplementary Figure 2:** The density of tumour vessels was assessed in PTs stained for collagen type IV (a: PT of a mouse treated with 500 ng ML-I/kg, b: PT of the control group). PTs treated with high dose ML-I (500 ng/kg body weight) showed a significant denser vascularisation than those of the control group (c).

**Supplementary Figure 3:** Primary tumours of all groups showed large necrotic areas (arrows).

**Supplementary Figure 4:** No significant differences in the dimension of necrotic tumour areas between the different groups were evident.
